# Complete Genome Sequence of an *Ehrlichia minasensis* Strain Isolated from Cattle

**DOI:** 10.1128/MRA.00161-19

**Published:** 2019-04-11

**Authors:** Daniel M. Aguiar, João P. Araujo, Luciano Nakazato, Emilie Bard, Alejandro Cabezas-Cruz

**Affiliations:** a Laboratory of Virology and Rickettsiosis, Veterinary Hospital (HOVET), Faculty of Veterinary Medicine (FAVET), Federal University of Mato Grosso (UFMT), Cuiabá, MT, Brazil; b Institute for Biotechnology, São Paulo State University (UNESP), Botucatu, SP, Brazil; c Laboratory of Molecular Biology, Veterinary Hospital (HOVET), Faculty of Veterinary Medicine (FAVET), Federal University of Mato Grosso (UFMT), Cuiabá, MT, Brazil; d EPIA, INRA, VetAgro Sup, Saint Genès Champanelle, France; e UMR BIPAR, INRA, ANSES, École Nationale Vétérinaire d’Alfort, Université Paris-Est, Maisons-Alfort, France; University of Maryland School of Medicine

## Abstract

Ehrlichia minasensis is a tick-borne pathogen affecting cattle, cervids, and dogs, and it is closely related to the monocytotropic pathogen Ehrlichia canis. Here, we announce the draft genome sequence of *Ehrlichia minasensis* strain Cuiabá, isolated from a naturally infected calf from Santo Antônio do Leverger, Mato Grosso, Brazil.

## ANNOUNCEMENT

The genus Ehrlichia is composed of tick-borne obligate intracellular Gram-negative alphaproteobacteria of the family Anaplasmataceae that primarily infect dogs (Ehrlichia canis, Ehrlichia chaffeensis, and Ehrlichia ewingii), mice (Ehrlichia muris), and ruminants (Ehrlichia ruminantium) (1). Some *Ehrlichia* species, however, have also been found to infect humans (i.e., *E. canis*, *E. ruminantium*, *E. chaffeensis*, and *E. ewingii*) (2, 3). Recently, we reported the *in vitro* culture (4), molecular (5), and morphological (6) characterization of *Ehrlichia minasensis* strain UFMG-EV^T^ (7), a novel *Ehrlichia* species that appears to have evolved from a highly variable strain of *E. canis* (8). Another *E. minasensis* strain (UFMT-BV) was found to be pathogenic for cattle in Brazil (9). The geographical distribution of *E. minasensis* is not restricted to the American continent as previously thought (5, 9, 10), and recent reports found these bacteria in Corsica, France (11), Pakistan (12), Ethiopia (13), and Israel (14).

The 16S rRNA phylogenetic tree shows that *Ehrlichia* is a diverse genus (7), and despite recent improvements in bacterial genome sequencing technologies, only a few genomes are available for *Ehrlichia* species, including those of *E. chaffeensis*, *E. ruminantium*, *E. canis*, *E. muris*, and *E. minasensis* UFMG-EV^T^ (15). Here, we report the genome sequence of *E. minasensis* strain Cuiabá, isolated from whole blood of an 8-month-old naturally infected male Holstein calf. The bacteria were grown in DH82 cells. The sample was treated with 2.5 units of Benzonase (Sigma-Aldrich, MO, USA) and 2 units of Turbo DNase (Thermo Fisher Scientific, Waltham, MA, USA) following total DNA extraction using a QIAamp DNA kit (Qiagen, Hilden, Germany) and quantified using Qubit fluorometric quantification (Thermo Fisher Scientific). The library was made starting from 5 ng of DNA using the Nextera XT library preparation kit (Illumina, San Diego, CA, USA), and the mean insert size of the ﬁnal library was 450 bp. The library was then sequenced with a NextSeq 550 system using sequencing by synthesis (SBS) chemistry (Illumina). Read quality was controlled using Illumina’s cloud service BaseSpace. A total of 37 million reads passed the quality ﬁlter; reads averaged 150 bp in length and showed an average quality score above Q30 in more than 90% of the bases. *De novo* assembly was performed with all quality reads using SPAdes (16). Prior to assembly, the overlapping reads were merged with Fast Length Adjustment of SHort reads (FLASH) (17). The contigs were aligned with Mauve (18) to the *E. minasensis* UFMG-EV^T^ genome (GenBank accession number CDGH00000000). The assembly step returned 59 contigs and 1,361,734 bases with an *N*_50_ value of 694,769 and 29.72% G+C content. The genome annotation was run with Prokka (19) and revealed the presence of 1,020, genes, including 978 coding sequences (CDSs) and 42 RNA genes (rRNA, transfer-messenger RNA [tmRNA], and tRNA). Among these, 346 genes are related to proteins with enzymatic activity (with defined Enzyme Commission [EC] codes), 93 are involved in cellular process and signaling with 15 related to membrane proteins, 162 in information storage and processing with 38 related to replication, recombination, and repair, and 226 in metabolism with 56 related to energy production and conversion.

*E. minasensis* genomes provide an invaluable tool for studying the relation between “host shift” (i.e., the capacity of some pathogens to infect novel hosts) and vector promiscuity within the *Anaplasmataceae*. This idea is supported by two facts. First, while the common tick vector of *E. canis* is almost exclusively Rhipicephalus sanguineus, *E. minasensis* in mainly found in Rhipicephalus microplus (5, 20) but has been detected also in Hyalomma marginatum (11) and Hyalomma anatolicum (12). Second, while *E. canis* is mainly pathogenic for dogs, E. minasensis infects not only bovines (9, 10, 13) and cervids (21) but also dogs (14).

### Data availability.

The raw sequence reads have been submitted to the NCBI SRA under the accession number PRJNA478569, and the whole-genome shotgun project of the *Ehrlichia minasensis* strain Cuiabá has been deposited in DDBJ/EMBL/GenBank under the accession number QOHL00000000. Genome assembly data are available under the accession number GCA_004181775.
